# Prognostic value of adenosine stress cardiovascular magnetic resonance in patients with low-risk chest pain

**DOI:** 10.1186/1532-429X-11-37

**Published:** 2009-09-21

**Authors:** Stamatios Lerakis, Dalton S McLean, Athanasios V Anadiotis, Matthew Janik, John N Oshinski, Nikolaos Alexopoulos, Elisa Zaragoza-Macias, Emir Veledar, Arthur E Stillman

**Affiliations:** 1 Department of Medicine and Division of Cardiology, Emory University School of Medicine, 1365 Clifton Road NE, Suite AT-500-508, Atlanta GA, 30322, USA; 2 Department of Radiology, Emory University School of Medicine, Atlanta, GA, USA

## Abstract

**Background:**

Approximately 5% of patients with an acute coronary syndrome are discharged from the emergency room with an erroneous diagnosis of non-cardiac chest pain. Highly accurate non-invasive stress imaging is valuable for assessment of low-risk chest pain patients to prevent these errors. Adenosine stress cardiovascular magnetic resonance (AS-CMR) is an imaging modality with increasing application. The goal of this study was to evaluate the negative prognostic value of AS-CMR among low-risk acute chest pain patients.

**Methods:**

We studied 103 patients, mean 56.7 ± 12.3 years of age, with chest pain and no electrocardiographic evidence of ischemia and negative cardiac biomarkers of necrosis, who were admitted to the Cardiac Decision Unit of our institution. All patients underwent AS-CMR. A negative AS-CMR was defined as absence of all the following: regional wall motion abnormalities at rest; perfusion defects during stress (adenosine) and rest; and myocardial scar on late gadolinium enhancement images. The patients were followed for a mean of 277 (range 161-462) days. The primary end point was defined as the combination of cardiac death, nonfatal acute myocardial infarction, re-hospitalization for chest pain, obstructive coronary artery disease (>50% coronary stenosis on invasive angiography) and coronary revascularization.

**Results:**

In 14 patients (13.6%), AS-CMR was positive. The remaining 89 patients (86.4%), who had negative AS-CMR, were discharged. No patient with negative AS-CMR reached the primary end-point during follow-up. The negative predictive value of AS-CMR was 100%.

**Conclusion:**

AS-CMR holds promise as a useful tool to rule out significant coronary artery disease in patients with low-risk chest pain. Patients with negative AS-CMR have an excellent short and mid-term prognosis.

## Background

Acute chest pain is one of the most common reasons for presentation to the emergency department [1]. The main tools for the initial triage of patients include history, electrocardiogram, and cardiac biomarkers of necrosis, which identify a large number of patients who would require admission and further management. The real challenge in contemporary chest pain units is the identification of patients with low-risk chest pain who could safely be discharged because on one hand, the "inappropriate" admission of these patients would pose a great burden of cost, while on the other, missing a diagnosis of chest pain of cardiac origin is associated with increased risk for adverse cardiac events in follow-up [2]. Furthermore, such a missed diagnosis is a frequent reason for malpractice claims [3]. Several advances in patients' triage, including new non-invasive imaging techniques [4] such as dobutamine stress echocardiography, stress nuclear studies, computed tomography coronary angiography and, recently, stress cardiovascular magnetic resonance (CMR), have enhanced the accuracy and efficiency of evaluation of patients with acute chest pain [5]. Highly desirable features of such techniques include a high negative prognostic value, i.e. a negative study would allow the safe discharge of patients with low-risk chest pain. The efficacy, however, of stress CMR in this setting has yet to be adequately explored. The aim of the present study was to assess the negative prognostic value of adenosine stress CMR (AS-CMR) in patients presenting to the emergency department with low-risk acute chest pain.

## Methods

### Study population

From July 2007 through May 2008, we enrolled 103 patients who presented to the emergency department with low-risk acute chest pain. This was a single center, retrospective study. All patients were examined clinically and cardiovascular risk factors were assessed, including tobacco use, diabetes mellitus, hypertension, hypercholesterolemia, family history of coronary artery disease (CAD) and history of previous CAD. Previous CAD was defined as previous myocardial infarction, CAD on cardiac catheterization, previous percutaneous coronary intervention or previous coronary artery bypass grafting. All cardiovascular medications were recorded. Low-risk acute chest pain was specified as no persistent ischemic-type chest pain with no objective evidence of myocardial ischemia, based on the negative results of serial serum cardiac biomarkers of necrosis (troponin I, MB fraction of creatinine kinase) as well as normal or inconclusive electrocardiograms (not indicative of ST-segment elevation myocardial infarction and without ≥2 mm T-wave inversion or >0.5 mm ST-segment depression). The exclusion criteria were standard contraindications for magnetic resonance imaging, such as internal pacemaker or defibrillator, cerebral aneurysm clips or metal in the eye; contraindications for adenosine administration, such as bronchospasm, high degree atrioventricular block, known allergic reaction to the medicine; pulmonary embolism; acute neurological disease; high clinical suspicion of acute aortic syndrome; and New York Heart Association class IV congestive heart failure symptoms.

All patients underwent AS-CMR within 24 hours of their presentation to the emergency department. This study had institutional review board approval from Emory University.

### AS-CMR protocol

**Figure 1:**
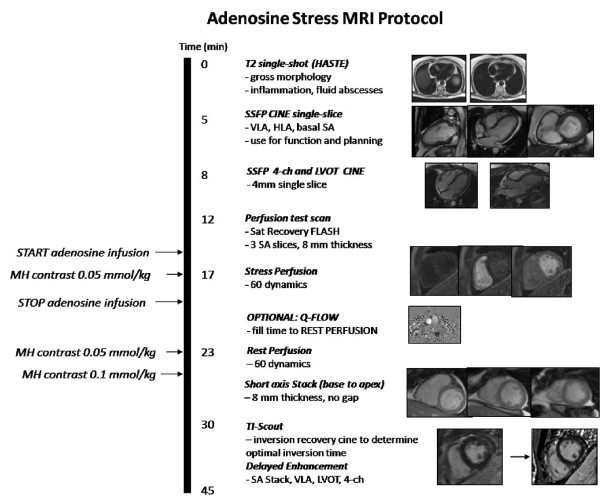
**The AS-CMR protocol**.

The AS-CMR protocol was carried out on a 1.5T Siemens Avanto system (Erlangen, Germany). The patient was positioned feet-first supine, with a 6-channel flexible body phased array coil anteriorly and a 24-element spine array for posterior signal reception. Three-lead ECG was applied to the chest with waveform monitoring throughout the study. The entire imaging protocol was completed in approximately 45 minutes (Figure 1).

Initial localization of the cardiac chambers was achieved using a T2-weighted single-shot fast spin echo (HASTE) sequence. Subsequently, breath-hold steady-state free precession (SSFP) cine acquisitions were acquired in the vertical long-axis, horizontal long-axis, basal short-axis, true four-chamber, and left ventricular outflow track orientations. Each orientation was prescribed using the preceding acquisition. First-pass perfusion (both at stress and rest) was performed with a saturation-prepared turbo flash acquisition. Three slices were oriented in the short-axis plane (base, mid, apex), prescribed from the cine four-chamber view. A test scan was performed with 5 dynamic images to confirm absence of artifacts and proper visualization of the LV myocardium and blood pool. The sequence was copied and 60 dynamics were implemented for perfusion imaging. Adenosine was administered intravenously at 140 μg/kg/min. Four minutes into the infusion, the stress perfusion imaging was performed, with 0.05 mmol/kg gadobenate dimeglumine (MultiHance), Bracco Diagnostics Inc., Princeton, NJ. The perfusion pulse sequence was initiated 3 seconds prior to gadolinium infusion, followed by a breath-hold command, and lasted for approximately 1 min. Adenosine infusion was stopped after the acquisition. After a brief period, during which optional images, such as flow sequences, were obtained, rest perfusion was performed with a second dose of 0.05 mmol/kg MultiHance, using the same acquisition method as was used for stress perfusion imaging. An additional 0.1 mmol/kg of MultiHance contrast was administered following rest perfusion for late gadolinium enhancement (LGE) imaging. Multi-slice SSFP cine short-axis imaging was subsequently performed. Acquisition extended from the atrial side of the ventricular valve plane to just beyond the apex. Approximately seven minutes after the final contrast dose, a multi-phase inversion-recovery SSFP scan was acquired in the mid short-axis plane, in order to determine the optimal inversion time (TI) to null normal myocardium for LGE imaging. The time that corresponded to the image with sufficiently nulled myocardium was entered as the TI into the subsequent inversion recovery segmented turbo flash sequence used for LGE imaging. Acquisition was performed in the short-axis, and covered the same anatomic range as the SSFP cine short-axis stack. In addition, single slices were acquired in the vertical long-axis, four chamber and left ventricular outflow tract views to correspond to the cine acquisitions. The TI was increased as needed as time passed.

Left ventricular ejection fraction was calculated off-line from end-diastolic and end-systolic endocardial tracings of the multi-slice SSFP cine short-axis images using computer-assisted planimetry. A negative AS-CMR was defined as the absence of all of the following: resting wall motion abnormalities on cine images, perfusion defects during adenosine infusion, or LGE in a coronary disease-type pattern (subendocardial enhancement). A positive AS-CMR had one or more of these features. A myocardial perfusion defect was reported if a segment was definitely darker than surrounding myocardium and persisted more than three images beyond initial peak enhancement of the segment which appeared most normal. LGE images were displayed with a gray scale to optimally show normal myocardium as dark and the regions of LGE or fat as bright. The AS-CMRs were interpreted qualitatively by consensus of two Level III CMR trained cardiologists or radiologists.

### Follow-up

The follow-up was completed in October 2008. The primary end point was defined as the combination of cardiac death, nonfatal myocardial infarction, re-hospitalization for unstable angina, obstructive CAD (defined as at least one coronary lesion of >50% stenosis on invasive coronary angiography) and coronary revascularization. Cardiovascular events during follow-up were identified for these patients by reviewing their medical data from our comprehensive institutional electronic records for clinic visits, new emergency department visits or hospital admissions. Myocardial infarction was defined based on the 2007 ESC/ACCF/AHA/WHF expert consensus document for the universal definition of myocardial infarction [6]. Cardiac death was defined as death associated with known or suspected acute myocardial infarction, life threatening arrhythmia, or cardiogenic pulmonary edema. Re-hospitalization for chest pain was defined as a recurrent episode of chest pain requiring hospital admission.

### Statistical analysis

Continuous variables are presented as mean ± SD or median (range). Qualitative variables are presented as absolute and relative frequencies. All analysis used a two tailed p value with a significance level of < 0.05. Statistical analyses were performed using the SAS statistical package version 9 (SAS Institute, North Carolina, USA).

## Results

During the 13 months of enrollment, 103 patients, 56.7 ± 12.3 years of age, with low-risk chest pain were included as study patients. Patients' characteristics, demographic data and medications at baseline are listed in Table 1. AS-CMR was performed in all cases without adverse events. The median duration of follow-up was 277 days (range 161 to 462 days). All 103 cases were followed up. There was no myocardial infarction, cardiovascular death or death from other cause in the study population.

**Table 1:** Baseline characteristics of the study population (n = 103).

Age, years	56.7 ± 12.3
Male gender	38 (36.9%)

**Risk factors**	

Hypertension	66 (64.1%)

Hypercholesterolemia	40 (38.8%)

Diabetes mellitus	30 (29.1%)

Smoking	20 (19.4%)

Family history of CVD	53 (51.5%)

Previous CAD	13 (12.6%)

**Medications**	

Acetylsalicylic acid	30 (29.1%)

ACEi/ARBs	35 (34.0%)

beta-blockers	31 (30.1%)

Calcium channel blockers	19 (18.4%)

Categorical variables are presented as absolute numbers and relative frequencies; continuous variables are presented as mean ± SD.

ACEi: angiotensin converting enzyme inhibitors; ARBs: angiotensin receptor blockers; CAD: coronary artery disease; CVD: cardiovascular disease.

A total of 14 patients (13.6% of the study population) had abnormal findings on AS-CMR. Six of these 14 patients (5.8%) had resting regional wall motion abnormalities, ten patients (9.7%) had perfusion defects during stress, and five patients (4.9%) had evidence of scar on LGE images. Six of the patients with an abnormal AS-CMR had a previously known history of CAD. Four patients refused further evaluation. From the remaining ten, five were advised to undergo coronary angiography, and all of these had obstructive CAD (one received percutaneous coronary intervention). Another four of the ten had known coronary anatomy from recent coronary angiography, and one with only a small area of ischemia was managed conservatively without undergoing coronary angiography. All patients received standard pharmacological treatment for secondary prevention of CAD. During the follow-up period, the conservatively-managed patient with a small area of ischemia presented again with chest pain and was admitted; coronary angiography was performed revealing luminal irregularities with no need for interventional treatment. Another two patients with known CAD and scar in the original AS-CMR were readmitted because of chest pain and underwent coronary angiography, with no need for revascularization. There were no readmissions for the patients who refused coronary angiography.

The remaining 89 patients (86.4% of the study population) had negative AS-CMRs. Seven of them had a known history of CAD. All patients were discharged from the emergency department with risk modification counseling and/or pharmacological treatment. During the period of follow-up a total of 9 patients presented again to the emergency department, all of them with atypical chest pain. Evaluation of chest pain with serial electrocardiograms, serial cardiac biomarkers of necrosis and non-invasive imaging studies did not reveal any case of acute coronary syndrome and all patients were discharged from the emergency department, without being admitted. The negative predictive value of AS-CMR was 100%.

## Discussion

The present study has shown that a negative AS-CMR performed within 24 hours of patients presenting to the emergency department with low-risk chest pain, as indicated by negative serial electrocardiograms and cardiac biomarkers of necrosis, is associated with an excellent prognosis.

Approximately 5% of patients with an acute coronary syndrome are discharged from the emergency room with an erroneous diagnosis of non-cardiac chest pain [7]. These patients are at substantial risk for subsequent cardiac events, as up to 15% will have a myocardial infarction in the subsequent two months [2]. Therefore, non-invasive tests which reliably exclude significant CAD in the acute setting are of great value, as they have the potential to reduce inappropriate admissions without increasing the risk of missed diagnoses.

### Prior Stress CMR Protocols

The present study extends the findings of previous studies examining the predictive value of stress perfusion CMR [8-15]. Jahnke et al. evaluated a combined sequential adenosine/dobutamine stress CMR imaging protocol, and showed that in a moderate risk population referred for ischemic evaluation a perfusion defect on AS-CMR was the best univariate predictor of death and non-fatal myocardial infarction [13]. In addition, a normal stress CMR predicted a low annual cardiac event rate in the range of 1%. However, their stress protocol was not solely employed in the acute setting and presenting symptoms included dyspnea as well as chest pain.

Also in the non-acute clinical setting, Pilz et al. [14] have shown that a normal AS-CMR predicts an excellent 1-year outcome. However, their study population also had varied indications for stress testing, with only 49% of patients presenting with angina; limiting its applicability to low risk patients presenting with acute chest pain. In addition, only patients without perfusion defects or LGE were included, leaving outcomes to be compared to calculated event probabilities rather than observed outcomes of a group with positive AS-CMR.

The high negative predictive value of AS-CMR found in the present study in patients presenting to the emergency department with low-risk chest pain concurs with findings from a prior study by Ingkanisorn et al [15], in which no patient with a normal AS-CMR had a subsequent diagnosis of CAD or an adverse outcome over a period of 467 days. While inclusion criteria were similar, all patients in the current study underwent AS-CMR from our Cardiac Decision Unit within 24 hours, whereas patients in Ingkanisorn et al. waited up to 72 hours with some patients presumably admitted to the hospital. Our study better validates the utilization of AS-CMR as an initial risk-stratifying diagnostic modality in the acute setting, selecting those patients who can be safely discharged. Also, only three short-axis slices were used for first-pass perfusion in the present study, compared with the "typically at least nine" slices obtained in Ingkanisorn et al. Therefore, our protocol maintains the same diagnostic accuracy with a less labor intensive image acquisition protocol.

### Comparison with Other Stress Modalities

Choosing the appropriate non-invasive evaluation for acute chest pain involves considering test characteristics, as well as the risks inherent to the test itself. High sensitivity and negative predictive value, as demonstrated for AS-CMR in the present study, are essential. For example, while several studies have shown that patients presenting to the emergency department with low-risk chest pain can safely undergo exercise testing within 6 to 12 hours, sensitivity of exercise treadmill alone for detection of CAD may be as low as 29% [16]. Adding nuclear myocardial perfusion imaging (MPI) to exercise protocols or pharmacologic stress improves sensitivity for detecting significant CAD, but results in significant exposure to ionizing radiation. Similarly, coronary computed tomography angiography (CTA) has a defined role in excluding CAD as the etiology of acute chest pain [17-20] but also involves radiation exposure and the administration of potentially nephrotoxic contrast agents. While efforts to decrease the effective radiation dose of these studies are making headway, the attributable risk of fatal malignancy from a single nuclear MPI study or coronary CTA has been estimated at 1 in 2000 [21].

Stress echocardiography is an attractive alternative for the evaluation of acute chest pain as it avoids exposure to radiation or nephrotoxic contrast agents, and includes an evaluation of cardiac structure and function similar to AS-CMR. However, the sensitivity of dobutamine stress echocardiography (DSE) with regional wall motion analaysis (WMA) for detection of significant CAD has been questioned, and its risks of induced arrhythmia and acute infarction are appreciable. Tsutsui et al. [22] suggest contrast administration with myocardial perfusion analysis (MPA) is necessary to achieve sufficient sensitivity to reliably exclude CAD. However, MPA is a technique not widely utilized. Contrary to these findings, Bedetti et al. [23] demonstrated the ability of normal WMA on pharmacologic stress echocardiogram to exclude significant CAD in a low risk population presenting with acute chest pain; as their subsequent coronary event rate was only 1.2% over a median follow-up of 13 months. A definitive comparison of stress echocardiography to alternative modalities has yet to be performed. Even if proven effective, it is estimated that 8% of DSEs done without contrast are not of diagnostic quality due to poor acoustic windows [24].

In addition to its high diagnostic accuracy and negative predictive value, AS-CMR is a safe and well tolerated procedure with only minimal risk to the patient. Due to the extremely short half-life of adenosine any adverse reactions caused by its infusion are rapidly ended with discontinuation of the drug. In the present study, there were no adverse effects associated with AS-CMR. Gadobenate dimeglumine carries a very low risk of nephrotoxicity, except in patients with end stage renal disease [25]. The imaging protocol itself was facile for a variety of technologists to apply with uniformly excellent image quality, and all studies were completed within the prescribed 45 minutes of cumulative scan time.

AS-CMR has specific advantages over other imaging modalities in addition to the avoidance of radiation, iodinated contrast, and dobutamine with its known risks. Unlike other imaging modalities, body habitus does not affect AS-CMR image quality nor the propensity for false positive results. In addition, alternative etiologies of chest pain including aortic dissection, pericarditis, and myocarditis may be accurately identified on AS-CMR. Furthermore, gold standard quantification of ejection fraction as well as assessment of valvular function are inherent to the exam. Finally, AS-CMR images can be read immediately and clinical decisions made quickly, as there is no post-processing required. This may reduce average length of stay in chest pain units, increase diagnostic efficiency, and allow practitioners to care for more patients.

### Subpopulations

The results of our study may have specific implications for women. Although usually underrepresented in studies of acute coronary syndromes, females were the majority of our population (63%). This may have partly accounted for the low incidence of adverse events in our study population and for the excellent negative predictive value of AS-CMR. Other forms of stress testing encounter specific obstacles among women, including low specificity for exercise testing and lower diagnostic accuracy for nuclear perfusion imaging due to the presence of breast artifact [26]. Furthermore, there is a greater risk of cancer development after radiation exposure (i.e. with computed tomography coronary angiography or stress nuclear imaging), especially among young women [27]. AS-CMR lacks all of these drawbacks.

The excellent negative predictive value of AS-CMR in our study population applied to patients both with and without a known history of CAD, although few patients had known CAD (7 out of 89 patients, 7.8%). According to current recommendations, patients with known CAD and low-risk chest pain can be observed in a chest pain unit and discharged if they have a negative stress test [17]. AS-CMR may help to distinguish CAD patients at high risk for future cardiac events from those with low risk and to guide proper management of these patients.

### Limitations

Our study has several limitations. First, this was a single-center study with small sample size resulting in the absence of a large number of hard cardiac events such as myocardial infarction or cardiac death. Secondly, we did not compare AS-CMR with other non-invasive or invasive imaging modalities. Third, follow-up was by review of medical records rather than directly contacting the patients. Furthermore, we calculated the negative but not the positive predictive value of AS-CMR. We believe that the intensive preventive management strategies in our positive AS-CMR patients could have accounted for a reduction in their risk; thus, the positive predictive value of AS-CMR would have been underestimated. Furthermore, a high negative predictive value is the most valuable characteristic for stress imaging in this clinical setting.

## Conclusion

Our study suggests that AS-CMR has high negative predictive value and can be used as a non-invasive stress imaging modality to safely discharge low-risk chest pain patients from the emergency department. Additional multicenter, large-scale studies could provide further insights into this area.

## Competing interests

The authors declare that they have no competing interests.

## Authors' contributions

SL conceived of the study and helped draft the manuscript. DSM helped gather data and helped draft the manuscript. MJ edited and revised the manuscript. JNO provided critical input on study design, and technical support for image acquisition. NA assisted with data analysis and statistics. AVA helped design the study and helped draft the manuscript. EZ assisted with statistical analysis and study design. EV assisted with study design. AES helped conceive the study and gather the data. All authors read and approved of the final manuscript.
